# “Language barriers and patient safety in supportive care: Two case reports bridging system-level gaps”

**DOI:** 10.1017/S1478951525101442

**Published:** 2026-01-19

**Authors:** Gladys Janice Jimenez-Torres, Monica Agosta, Miriam Garcia-Hocker, Ahsan Azhar, Eduardo Bruera, Cindy Carmack

**Affiliations:** Department of Palliative Care, Rehabilitation, and Integrative Medicine, The University of Texas MD Anderson Cancer Center, Houston, TX, USA

**Keywords:** Palliative psychology, supportive care, non-English language preference, health equity, discharge safety

## Abstract

**Objectives:**

Patients with non-English language preference (NELP) face significant barriers to safe and effective communication in palliative and supportive care. These barriers compromise quality, delay care, and heighten the risk of unmet psychosocial needs, particularly when compounded by health literacy concerns and limited access to translated resources.

**Methods:**

We describe two Spanish-speaking patients with advanced cancer whose inpatient and outpatient supportive care was complicated by language barriers, leading to communication gaps, including symptom misinterpretation and inadequate family discharge education. These factors contributed to significant distress and safety risks.

**Results:**

Interdisciplinary interventions, including professional interpreter use, bilingual supportive care psychology, teach-back education, medication relabeling in Spanish, and culturally tailored communication, helped restore trust, alleviate suffering, and align care with patient and family values. In one case, a lack of validated Spanish versions of the Edmonton Symptom Assessment System within the electronic medical record (EMR) hindered symptom self-reporting and safety. Following multiple requests, the EMR team initiated development of a Spanish-language template to facilitate future integration.

**Significance of the results:**

Structural gaps in language accessibility compound distress, reduce autonomy, and threaten safety. These cases underscore that interpreter services alone are insufficient. Integrating validated multilingual tools into EMRs, standardizing translated discharge instructions, and expanding access to in-person interpreters are critical steps toward equitable care. Institutionalizing linguistically responsive systems is essential for ensuring safety, equity, and dignity in palliative care for patients with NELP.

## Introduction

Language barriers significantly compromise equity, safety, and outcomes in palliative care (Silva et al. 2016; Barwise et al. 2019; Fox et al. 2020; Bigger et al. 2025). In the United States, over 22% of people aged five or older speak a language other than English at home, with Spanish being the most common (Bureau USC 2025). Patients with non-English language preference (NELP) often experience disparities in communication, symptom management, and care transitions (Abedini et al. 2022; Schwartz et al. 2022; Escobedo et al. 2023; Smith et al. 2024). In oncology, for example, among patients with advanced pancreatic cancer, NELP was suggested to contribute to worse clinical and survival outcomes, with evidence suggesting that NELP itself may be an independent risk factor (Williams et al. 2024).

Reliance on interpreter services alone rarely addresses these complexities, particularly when compounded by limited health literacy, lack of standardized translated resources, and the inherent emotional burden of advanced disease (Lehman and Moriarty 2024; Yuen et al. 2024). In a recent national survey of 1,660 medical interpreters, Howard et al. (2025) reported that 70.4% seldom or never met with medical providers prior to goals-of-care (GOC) discussions, while nearly 75% expressed interest in receiving training on end-of-life (EOL) topics and communication. These findings highlight a critical gap in interdisciplinary preparedness, emphasizing the need for structured collaboration and shared education between interpreters and clinical teams in palliative care. Differences across care modalities further compound inequities, as populations with NELP face variable access to inpatient versus outpatient services, leading to poorer overall outcomes (Twersky et al. 2024). Interpreter-related challenges also arise as professional interpreters may struggle with the emotional weight of palliative care discussions, while family members serving as interpreters risk introducing ethical dilemmas that compromise accuracy and autonomy (Krystallidou et al. 2017; Latif et al. 2022; Hancox et al. 2023). Although standardized protocols, such as order sets for EOL symptoms management, help mitigate some disparities, systemic inequities persist (Curatola et al. 2024).

We present two cases involving Spanish-speaking patients with advanced cancer whose care was complicated by systemic language barriers. These cases demonstrate that NELP is only partially addressed by the availability of interpreter services. Gaps in electronic medical record (EMR) integration, non-standard bilingual documentation, and the absence of validated Spanish tools can compromise safety and the quality of care. Clinician-level interventions, such as language-congruent communication, utilizing culturally informed psychological support to address acculturative stress, and bilingual educational strategies with a supportive care psychologist leading interdisciplinary efforts, can help to mitigate inequities.

## Case reports

### Case 1

A 51-year-old woman with a rare form of metastatic carcinoma was admitted for uncontrolled abdominal pain and complications from a jejunostomy (J-tube) leak. She had previously received FOLFOX chemotherapy and J-tube placement and was referred to supportive care psychology for anxiety, sadness, and grief. During her final inpatient session with a bilingual psychologist, she and her parents expressed confusion and distress about her discharge instructions.

They reported difficulty accessing adequate virtual translation services due to poor internet connectivity and were unable to read discharge paperwork with instructions utilizing a mix of English and Spanish, with medication labels entirely in English. The patient and her family’s limited health literacy compounded the problem; they had received minimal hands-on training for J-tube and nephrostomy care and noted discrepancies in the written instructions provided.

With the bilingual supportive care psychologist’s help, the interdisciplinary team members were recruited to implement corrective interventions. Teach-back and experiential learning were used to provide J-tube and percutaneous nephrostomy catheter care, opioid safety education was provided in Spanish, and the advanced practice nurse for her primary team updated the EMR to reflect Spanish language preference for any shared documents. Simultaneously, the charge nurse coordinated with pharmacy to relabel medications in Spanish while the psychologist (with support from her primary nurse) annotated medication lists with Spanish explanations.

By discharge, the patient’s mother, who initially was fearful and tearful, expressed gratitude and confidence in managing her daughter’s care at home. Consistent verbal and written communication in their preferred language proved essential in reducing distress and restoring trust.

### Case 2

A 65-year-old woman with metastatic ovarian cancer and peritoneal carcinomatosis, who was undergoing systemic therapy and awaiting surgery, presented with significant psychological distress related to family separation and treatment side effects. During outpatient follow-up with supportive care, she was asked to complete the Edmonton Symptom Assessment System (ESAS) (Carvajal et al. 2013). Although validated in Spanish, only the English version was available on the electronic platform, which the patient was required to complete before her visit. Without staff assistance prior to her session, she struggled and allowed her family to intervene and translate for her, compromising privacy and accuracy.

During supportive care psychology sessions conducted in Spanish, she expressed how dependence on others to navigate an English-only system intensified her sense of vulnerability. She reported elevated distress when having to answer questions about her symptom burden without full comprehension. Review of ESAS items with her bilingual psychologist during sessions helped her restore trust and ensure accurate responses, reducing the risk of over- or under-treatment. Despite multiple safety reports and Information Technology (IT) requests, the validated Spanish versions of the ESAS have not yet been incorporated into the EMR. Recently, however, the EMR team reported they had begun developing the template as the first step toward integration.

## Discussion

These cases illustrate how language barriers can compromise core elements of supportive care, including discharge safety, symptom assessment, and emotional well-being. In Case 1, fragmented translation, inconsistent bilingual documentation, limited self-care training, and English-only medication labels created significant anxiety for the patient and her family, nearly jeopardized discharge safety, and undermined their confidence in home management. Prior studies confirm that language discordance disrupts routine clinical tasks such as medication administration, pain management, and other routine nursing duties, and therefore increases safety risk for patients with NELP (Karliner et al. 2012; Silva et al. 2016; van Rosse et al. 2016). Moreover, patients with NELP frequently misunderstand medication categories and purposes, and inaccurate or untranslated labels pose measurable safety risks (Karliner et al. 2012). These factors further compound the decreased confidence in care that NELP patients are known to experience, in addition to exacerbating physical and emotional symptom burden (Lehman and Moriarty 2024).

In Case 2, the absence of validated multilingual assessment tools within the EMR limited independent symptom reporting and reinforced reliance on family members to navigate an English-only system. Because clinicians and medical teams rely on the ESAS to assess symptom burden, language-appropriate administration is critical to safety. When using screening tools, such as the ESAS, to assess issues of concern and degrees of distress, it is imperative to have accurate feedback from the patient’s perspective to prevent compromising safety. Prior work confirms that the validated Spanish versions of the ESAS and similar symptom screening tools exist but are inconsistently implemented across electronic platforms, impeding symptom self-report (Carvajal et al. 2013). This underscores a system-level failure rather than a patient-level deficit.

In both cases, NELP contributed to miscommunication that compromised patient safety and symptom management. These cases also required additional time invested by specialty clinicians, ensuring understanding, highlighting the burden placed on interdisciplinary teams and families when language accessibility is inadequate. Addressing these challenges required interdisciplinary collaboration and rapid and intentional interventions tailored to the patients’ needs. These included correcting records, relabeling medications, and translating education, as well as the use of the teach-back method and bedside return demonstrations. The teach-back method and stepwise experiential learning, supported by bilingual clinicians, improved comprehension and confidence (Choi and Choi 2021; Dewi et al. 2023; Tsui et al. 2023). Bilingual supportive care, psychology, and family involvement were critical in reducing distress.

Intentional efforts to match patients and families with language-concordant teams can help mitigate inequities arising from language and cultural barriers to high-quality care (Bregio et al. 2022). Nonetheless, institutional policies that restrict clinicians from using their native language in patient encounters (e.g., by requiring additional certification or testing) may inadvertently exacerbate communication barriers. For clinicians who are fully fluent and professionally educated in that language, such regulations can introduce unnecessary administrative obstacles that hinder direct communication and increase reliance on third-party interpreters. Revisiting these policies could help reduce avoidable complexity and promote more effective, person-centered care. These challenges underscore the limits of relying on individualized solutions, which are inconsistently feasible and risk perpetuating inequities when resources are limited (Escobedo et al. 2023; Twersky et al. 2024).

System-level changes are therefore essential to ensure long-term equity in care delivery. Integration of validated multilingual tools within EMR, expanded access to in-person interpreters, standardized translated discharge instructions, multiple reviews of medication labeling, and culturally sensitive, hands-on education can enhance safety and strengthen patients and their families’ confidence (Karliner et al. 2012; Davis et al. 2019). Educating interpreters on EOL topics and GOC discussions could also improve their ability to navigate these conversations and manage the emotional impact they carry (Howard et al. 2025). Embedding linguistically responsive practices into institutional quality improvement initiatives may also promote greater accountability at the leadership level. These practices might include translation audits, interpreter use metrics, patient-reported communication quality assessments, and tracking the impact of these initiatives through patient outcomes (Silva et al. 2016; Fox et al. 2020; Kung et al. 2023). Collaboration with community advisory groups representing populations with NELP in the revision of policies and practices could further ensure interventions at multilevel align with real-world needs (Ridgeway et al. 2021).

Although these cases focused on Spanish-speaking patients, the lessons apply broadly across languages and settings. Prioritizing linguistically responsive care as a standard of practice is necessary to protect safety, support autonomy, and maintain dignity. True equity requires system-level integration of validated multilingual tools, consistent interpreter availability, and culturally competent workflows, so that linguistically responsive care becomes the new norm rather than the exception.
